# Supplementary material for: Chat to chip: large language model based design of arbitrarily shaped metasurfaces
Source: Nanophotonics. 2025 Oct 9;14(22):3625–33. doi: 10.1515/nanoph-2025-0343 (PMC12592791; doi:10.1515/nanoph-2025-0343)
Supplement: Supplementary file 1 — Supplementary Material Details [file j_nanoph-2025-0343_suppl_001.docx]

**Supplementary Materials**

**Chat to Chip: Large Language Model Based Design of Arbitrarily Shaped Metasurfaces**

Huanshu Zhang,^1^ Lei Kang,^1^ Sawyer D. Campbell,^1^ and Douglas H. Werner^1, *^

^1^ Department of Electrical Engineering, The Pennsylvania State University, University Park, PA 16802, USA.

^*^Corresponding Author: [dhw@psu.edu](mailto:dhw@psu.edu)

**This SI includes:**

**1. More examples of inverse design that show distinction between target shapes and designed shapes**

**2. Tandem network results**

**3. Observations between tandem networks and LLMs for inverse design**

**1. More examples of inverse design**


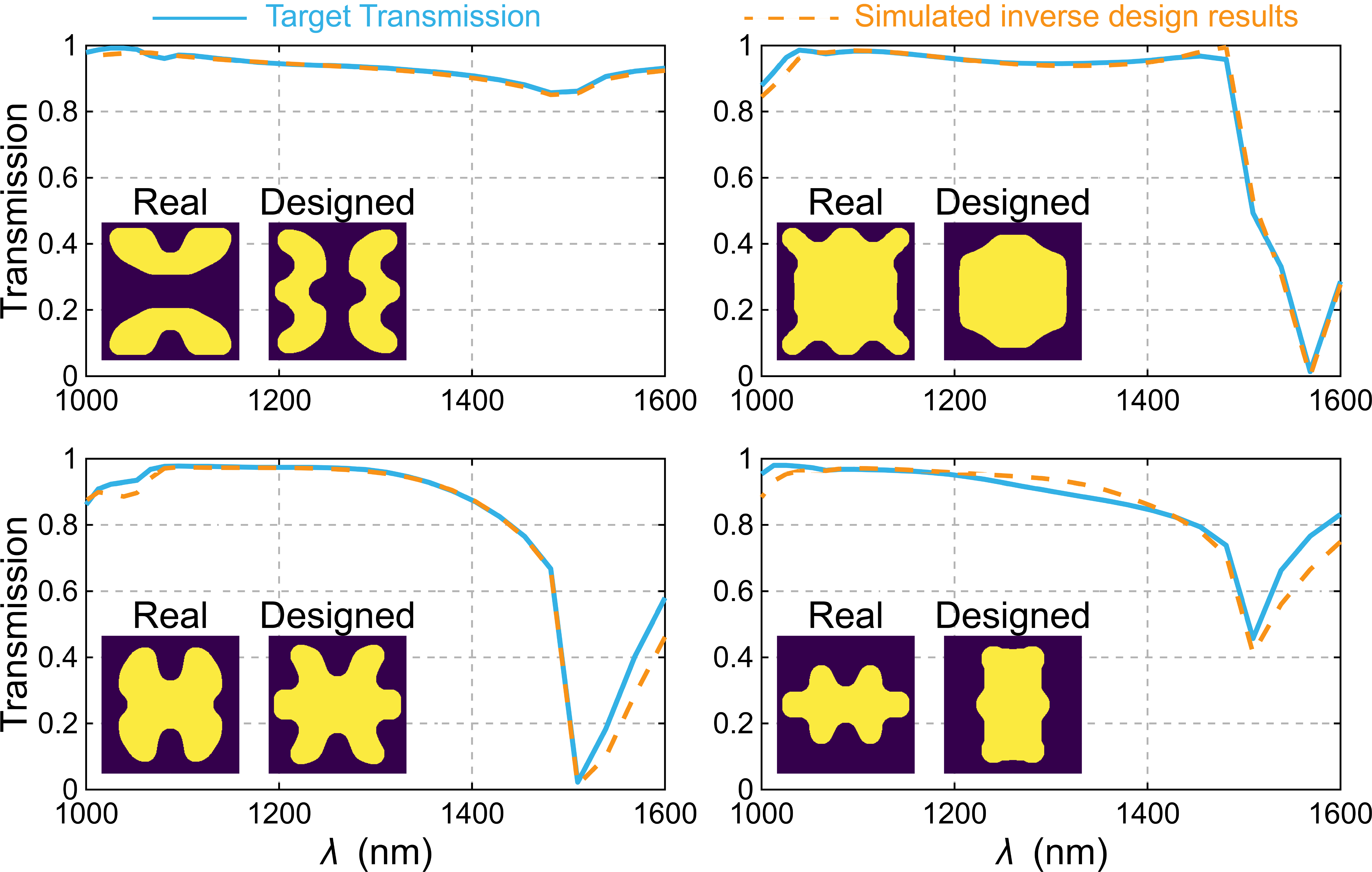


Figure S1. Representative results for four unseen targets. The orange dashed lines are FDTD simulated results of inverse-designed metasurfaces. The corresponding inverse-designed grids and MSE are: top-left: [[0.453, 0.285, 0.247, 0.896], [0.148, 0.960, 0.787, 0.045], [0.286, 0.335, 0.984, 0.063], [0.429, 0.859, 0.050, 0.453]], MSE = 6.6 × 10^-5^; top-right: [[0.200, 0.006, 0.426, 0.290], [0.714, 0.453, 0.600, 0.606], [0.206, 0.632, 0.584, 0.563], [0.024, 0.622, 0.182, 0.765]], MSE = 3.1 × 10^-4^; bottom-left: [[0.649, 0.506, 0.089, 0.160], [0.162, 0.589, 0.824, 0.082], [0.843, 0.396, 0.854, 0.593], [0.937, 0.792, 0.716, 0.887]], MSE = 1.3 × 10^-3^; bottom-right: [[0.803, 0.217, 0.334, 0.287], [0.139, 0.278, 0.606, 0.454], [0.607, 0.347, 0.479, 0.406], [0.319, 0.422, 0.691, 0.693]], MSE = 1.5 × 10^-3^. These results demonstrate that the LLM approach successfully mitigates the many-to-one non-convergence problem.

**2. Tandem network results**


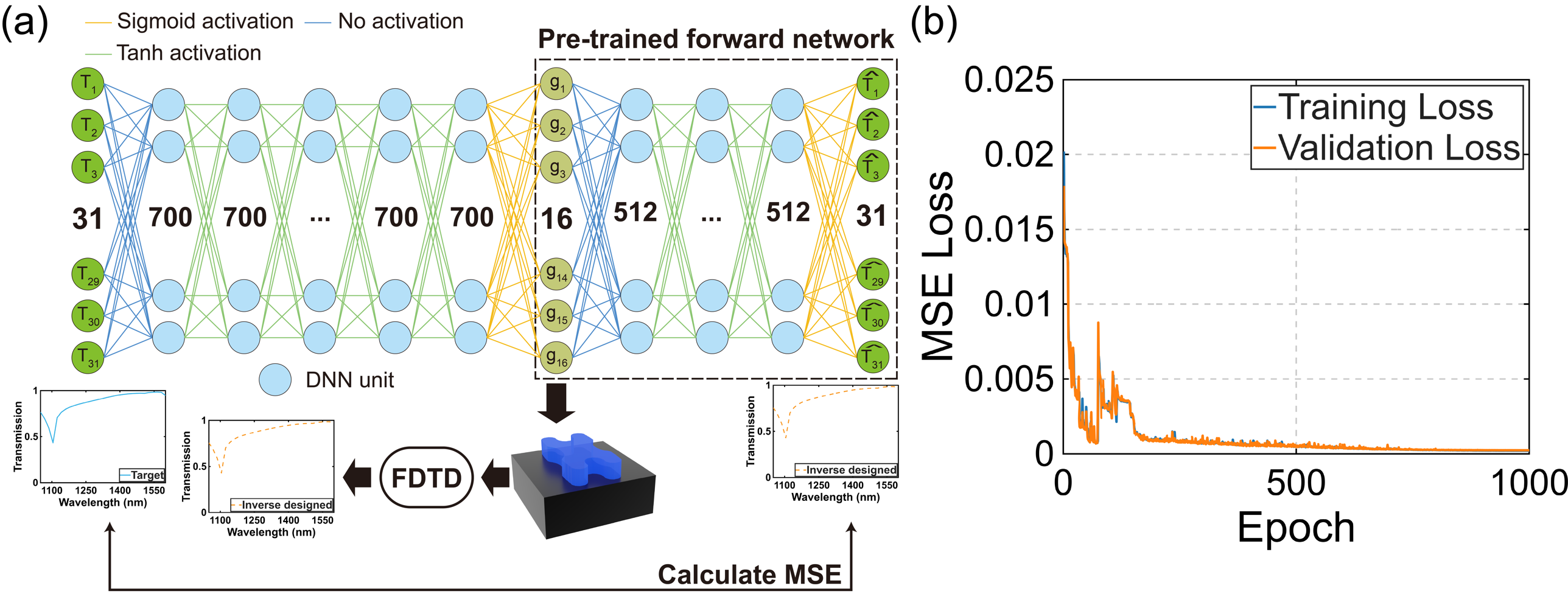


Figure S2. Tandem network structure and training loss. (a) A classical tandem network structure. Both forward and inverse networks are composed of multiple fully connected layers. The pre-trained forward network ingests the 4×4 control points flattened to a 16-dimensional vector, passes them through an initial fully connected expansion to 512 units, and then proceeds through a homogeneous stack of eight hidden layers, each of width 512, with a tanh applied after every hidden affine transformation; a final linear layer followed by a component-wise sigmoid yields a 31-dimensional spectral output constrained to [0, 1]. The MSE of this forward network on the test set is 2.0 × 10^-3^. Conversely, the inverse network accepts a 31-element spectrum, expands to 700 units, and traverses six hidden layers of width 700 with the same tanh activation after each hidden layer, concluding with a linear projection and component-wise sigmoid that returns a 16-dimensional vector corresponding to the flattened 4×4 control points, also bounded to [0, 1]. The loss function calculates the MSE between the target transmission and the predicted transmission and only uses this loss to update the weights of the inverse network while keeping the forward network unchanged. The final MSE′, which is defined as the MSE between the target transmissions and the predicted transmissions by the forward network, is 2.4 × 10^-4^. This figure is reproduced with permission from [Zhang, Huanshu, *et al.* "Fixed-attention mechanism for deep-learning-assisted design of high-degree-of-freedom 3D metamaterials," *Optics Express* 33.9 (2025): 18928-18937]. Copyright 2025 Optical Society of America. (b) Learning curve for the backward training process.





Figure S3. Representative results for some unseen targets. The orange dashed lines are FDTD simulated results of inverse-designed metasurfaces. (a) The corresponding inverse-designed grids and MSE are: top-left: [[1.000, 1.000, 1.000, 1.000], [1.000, 0.198, 0.305, 0.037], [0.622, 1.000, 0.284, 0.559], [0.222, 0.732, 1.000, 0.005]], MSE = 1.9 × 10^-5^, the MSE of the same design from LLM is 2.0 × 10^-7^; top-right: [[1.000, 1.000, 1.000, 1.000], [1.000, 0.127, 0.315, 0.008], [0.002, 1.000, 0.775, 0.0525], [0.268, 0.497, 1.000, 0.569]], MSE = 1.0 × 10^-4^, the MSE of the same design from LLM is 1.2 × 10^-6^; bottom-left: [[1.000, 1.000, 1.000, 1.000], [1.000, 0.391, 0.973, 0.024], [0.304, 1.000, 0.053, 0.588], [0.750, 0.931, 1.000, 0.758]], MSE = 7.4 × 10^-5^, the MSE of the same design from LLM is 1.4 × 10^-6^; bottom-right: [[1.000, 1.000, 1.000, 1.000], [1.000, 0.408, 0.554, 0.058], [0.699, 1.000, 0.218, 0.643], [0.281, 0.886, 1.000, 0.028]], MSE = 2.2 × 10^-4^, the MSE of the same design from LLM is 3.0 × 10^-7^. (b) The corresponding inverse-designed grids and MSE are: top-left: [[1.000, 1.000, 1.000, 1.000], [1.000, 0.376, 0.524, 0.048], [0.627, 1.000, 0.280, 0.604], [0.304, 0.875, 1.000, 0.030]], MSE = 6.0 × 10^-4^, the MSE of the same design from LLM is 6.6 × 10^-5^; top-right: [[1.000, 1.000, 1.000, 1.000], [1.000, 0.256, 0.520, 0.726], [0.177, 1.000, 0.983, 0.995], [0.108, 0.986, 1.000, 0.346]], MSE = 8.5 × 10^-4^, the MSE of the same design from LLM is 3.1 × 10^-4^; bottom-left: [[1.000, 1.000, 1.000, 1.000], [1.000, 0.237, 0.761, 0.074], [0.001, 1.000, 0.8710, 0.592], [0.017, 0.593, 1.000, 0.553]], MSE = 1.1 × 10^-3^, the MSE of the same design from LLM is 1.3 × 10^-3^; bottom-right: [[1.000, 1.000, 1.000, 1.000], [1.000, 0.224, 0.387, 0.011], [0.001, 1.000, 0.803, 0.144], [0.104, 0.503, 1.000, 0.835]], MSE = 1.8 × 10^-4^, the MSE of the same design from LLM is 1.5 × 10^-3^. These results demonstrate that the classical tandem network approach successfully mitigates the many-to-one non-convergence problem.

**3. Observations between tandem networks and LLMs for inverse design**

The LLM-based inverse designer exhibits greater solution diversity than the tandem baseline while lacking fidelity in certain structures. In the tandem setting, predicted control points frequently saturate at the upper bound (repeating 1.000 entries across large subblocks), which is characteristic of boundary clamping and partial mode collapse when an inverse network is trained solely through a frozen forward network under a bounded output layer. By contrast, the LLM produces interior-valued, heterogeneous grids that avoid clamping. The LLM’s spread of solutions is consistent with the intrinsically many-to-one nature of inverse metasurface design. Taken together, these observations indicate that the LLM preserves geometric degrees of freedom and achieves a favorable fidelity-diversity-AI-knowledge trade-off without collapsing to boundary solutions, qualities that are desirable for practical inverse design workflows.
